# Idelalisib improves CD37 antibody BI 836826 cytotoxicity against chemo-resistant /relapse-initiating CLL cells: a rationale for combination treatment

**DOI:** 10.1038/bcj.2016.106

**Published:** 2016-11-11

**Authors:** S Betrian, L Ysebaert, K H Heider, J P Delord, J J Fournié, A Quillet-Mary

**Affiliations:** 1 CRCT, Université de Toulouse, CNRS ERL 5294, Inserm U1037, Toulouse, France; 2 Department of Haematology, Institut Universitaire du Cancer, Toulouse, France; 3 Boehringer Ingelheim RCV GmbH & Co KG, Wien, Austria; 4 Institut Universitaire du Cancer, Bureau d'études cliniques, Toulouse, France

Chronic lymphocytic leukaemia (CLL) is characterized by the accumulation of CD5^+^/CD19^+^ B leukemic cells in blood and secondary lymphoid organs. Despite significant advances in its therapy, especially during the last two decades, the disease still remains incurable and new treatment options need to be developed. Currently, the CD20 antibody, rituximab, is used to treat CLL in combination with chemotherapy. The addition of rituximab to a fludarabine/ cyclophosphamide chemotherapy regimen led to a longer overall survival, defining the current treatment standard.^1^ However, rituximab efficacy is limited in the subgroup of patient with chromosome 17p deletion.^1^

In recent years, it has been demonstrated that CLL proliferative centers are mostly localized in lymph nodes,^2^ and that leukemic cells survival is extremely dependent on cross-talk with the tumor microenvironment.^3^ Indeed, interactions between B cells and chemokines, immune-suppressive cells in tumor environment, promote survival as well as chemotherapy resistance. Among chemo-resistant cells, side population (SP) cells have been reported in blood samples from CLL patients.^4, 5^ SP cells appear resistant to conventional treatment including fludarabine, bendamustine and rituximab, and seem to be selected by these treatments.^4, 5^ SP cells should be considered as relapse-initiating cells in the development of new therapeutic agents.

CD20 is not the sole tangible protein on leukemic B cells. Other antigens are also under investigation to develop therapeutic antibodies, that is, CD37. As a target antigen, the tetraspanin CD37 has a higher and homogeneous expression on CLL cells than CD20.^6^ The Fc-engineered CD37 antibody, BI 836826, showed remarkable direct cytotoxicity against isolated CLL cells and led to potent B-cell depletion from whole-blood samples.^7^ In this study, we assessed the efficacy of BI 836826 on global and chemo-resistant SP B-cell depletion from relapsed CLL patient's samples *ex vivo* (Supplementary Information 1). For this purpose, we collected samples from relapsed patients, mainly after FR/FCR treatment, and from a phase I clinical study with BI 836826 monotherapy for CLL patients. Fresh peripheral blood mononuclear cells (PBMC), isolated from blood samples by Ficoll gradient centrifugation, were subsequently cultured in high-density cultures (10 × 10^6^ cells per ml) allowing to work with viable cultures for >7 days.^8, 9^ SP cells experiments were done as previously described (Supplementary Information 3).^5, 10^ Flow cytometric analysis revealed a higher CD37 expression in SP cells compared with its non-SP counterpart in CLL samples from relapsed patients (Figure 1a), suggesting that SP cells could be sensitive to BI 836826 cytotoxicity. The PBMC depletion assays were then performed in a large cohort (*n=*16) of relapsed CLL patients. In agreement with published results,^11^ B leukemic cells were better depleted by BI 836826 treatment than by RTX (Figure 1b). We previously described that CLL SP cells were insensitive to RTX-induced cytotoxicity.^5^ Here no differences were observed between SP and non-SP cells in terms of BI 836826-induced B-cell depletion, suggesting that both populations were equally targeted by BI 836826 and that CD37 could be a better target to deplete CLL leukemic cells *in vivo* (Figure 1c).

In order to test this hypothesis, we evaluated SP and non-SP cell depletion in a phase I clinical study (NCT01296932) on BI 836826 monotherapy in four relapsed CLL patients. SP and non-SP cell populations were analysed during the treatment (Figure 1d). Despite a high variability in the response, we observed that, *in vivo*, BI 836826 induced not only non-SP cell depletion but also particularly SP cell depletion in 3 patients (75%). Since BI 836826 is able to target chemo-resistant/relapse-initiating CLL cells *in vivo*, our results underline the potential benefit of BI 836826 clinical efficacy in CLL treatment.

The CLL proliferation centers are located in secondary organs such as lymph nodes,^2^ in which poor bioavailability of the treatments decrease their efficacy. Thus, the understanding of molecular pathways involved in leukemic B-cell survival led to the development of new therapeutic targets. Among recent treatments, Idelalisib, an oral potent selective inhibitor of PI3kinase delta, inhibits survival network from microenvironment^12^ and significantly improves survival among patients with relapsed CLL.^13^ Indeed, Idelalisib was described to induce the egress of leukemic cells from lymph nodes to blood circulation for a better targeting.^13^ Idelalisib was shown to enhance RTX- or BI 836826-induced cytotocity in CLL samples *in vitro*.^14^ Moreover, the signalling pathway associated with CD37 ligation leads to several phosphorylations among PI3K delta recruitment, promoting cell survival.^15^ Thus, combination treatment with a PI3K delta inhibitor and CD37 antibody has a biological rationale and is currently investigated as a first-line treatment for CLL patients in a clinical trial (NCT02538614). In addition, comparative analysis between lymph node and blood samples^2^ revealed an enrichment of SP cells in lymphoid organs (Figure 2a). We hypothesized that Idelalisib might enforce the egress of these cells into the blood compartment making them vulnerable to BI 836826.

In order to test the efficacy of BI 836826/Idelalisib combination in SP and non-SP cells, we performed *in vitro* depletion experiments in fresh PBMC samples from relapsed CLL patients (Supplementary Information 1) with a suboptimal dose of Idelalisib (0.5 μM) in the presence or not of BI 836826 or BI 836847 (isotype control) (10 μg/ml). First we assessed that Idelalisib *in vitro* did not change CD37 expression (Supplementary Information 2a). We confirmed that the BI 836826/Idelalisib combination is significantly more cytotoxic than single agents in PBMC samples from relapsed CLL patients (Figure 2b). We then assessed BI 836826-induced B-cell depletion *ex vivo* in Idelalisib-treated patients (150 mg BID). Since Idelalisib induced a peak of lymphocytosis (*n=*10; Figure 2c), CD37 expression and B-cell depletion were quantified before treatment and at the peak of lymphocytosis. No changes in CD37 expression were observed at these two time points (Supplementary Information 2b). Depletion experiments showed that, Idelalisib treatment *in vivo* did not impair BI 836826 efficacy (Figure 2d). Five patients from the same cohort were analysed for SP and non-SP cell quantification as well as BI 836826 sensitivity. We observed that Idelalisib induced an increase of both non-SP and SP cells in blood. Noteworthy, fold change of SP cells reaching blood is twice as high as non-SP cells (Figure 2e). Finally, SP cells from Idelalisib-treated patients were significantly more sensitive to BI 836826-induced cytotoxicity (Figure 2f).

These findings suggest that BI 836826 targets leukemic B cells and chemoresistant SP cells in CLL patients with similar efficacy. Moreover, Idelalisib treatment disrupts microenvironment cell-cell interactions and drives SP cell egress from lymph nodes to the blood compartment. Based on biological rationale and the demonstrated *in vitro* and *ex vivo* efficacy, our data support the combination of Idelalisib and BI 836826 as a potential new therapeutic option in CLL treatment for relapsed/refractory patients.

## Figures and Tables

**Figure 1:**
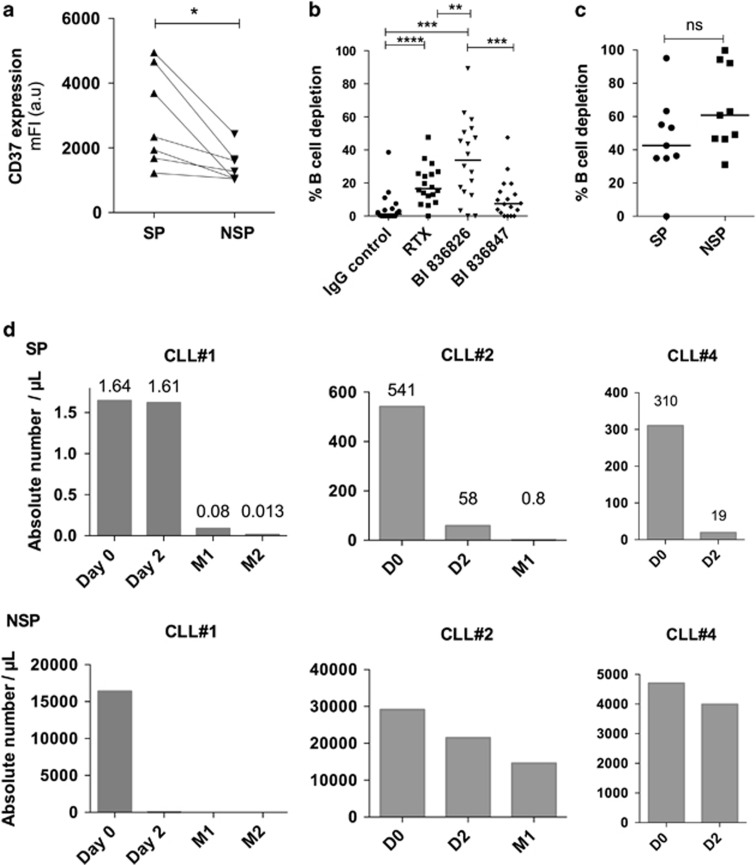
BI 836826 efficacy in SP and non-SP B leukemic cells from relapsed patients. (**a**) CD37 expression in SP and non-SP cells: cells were labeled with Hoechst 33342 in the presence or not of Verapamil^5, 10^ with control Isotype or FITC BI 836826 antibody (*n=*7). (**b**) Antibody-induced B-cell depletion: fresh PBMC were incubated in the presence of IgG control, RTX, BI 836826 or BI 836847 (isotype control) (10 μg/ml) for 7 days. Antibody-mediated B-cell depletion was determined by enumerating trypan blue-negative combined to CD5/CD19-positive B lymphocytes, determination by flow cytometry^7^ (*n=*18). (**c**) Antibody-induced B-cell depletion in SP and non-SP populations: fresh PBMC were incubated in the presence of 10 μg/ml BI 836826 or BI 836847 (isotype control) (10 μg/ml) for 7 days and then processed for SP and non-SP analysis and quantification (*n=*9). (**d**) SP and non-SP quantification in BI 836826-treated patients (*n=*3). Flow cytometric experiments were done on a FACS BDLSR2 or FORTESSA X20 (Becton Dickinson) and analysed by DIVA software. Statistical analysis: paired Student *t*-test. **P<0.001; ***P<0.005; ****P<0.0001.

**Figure 2:**
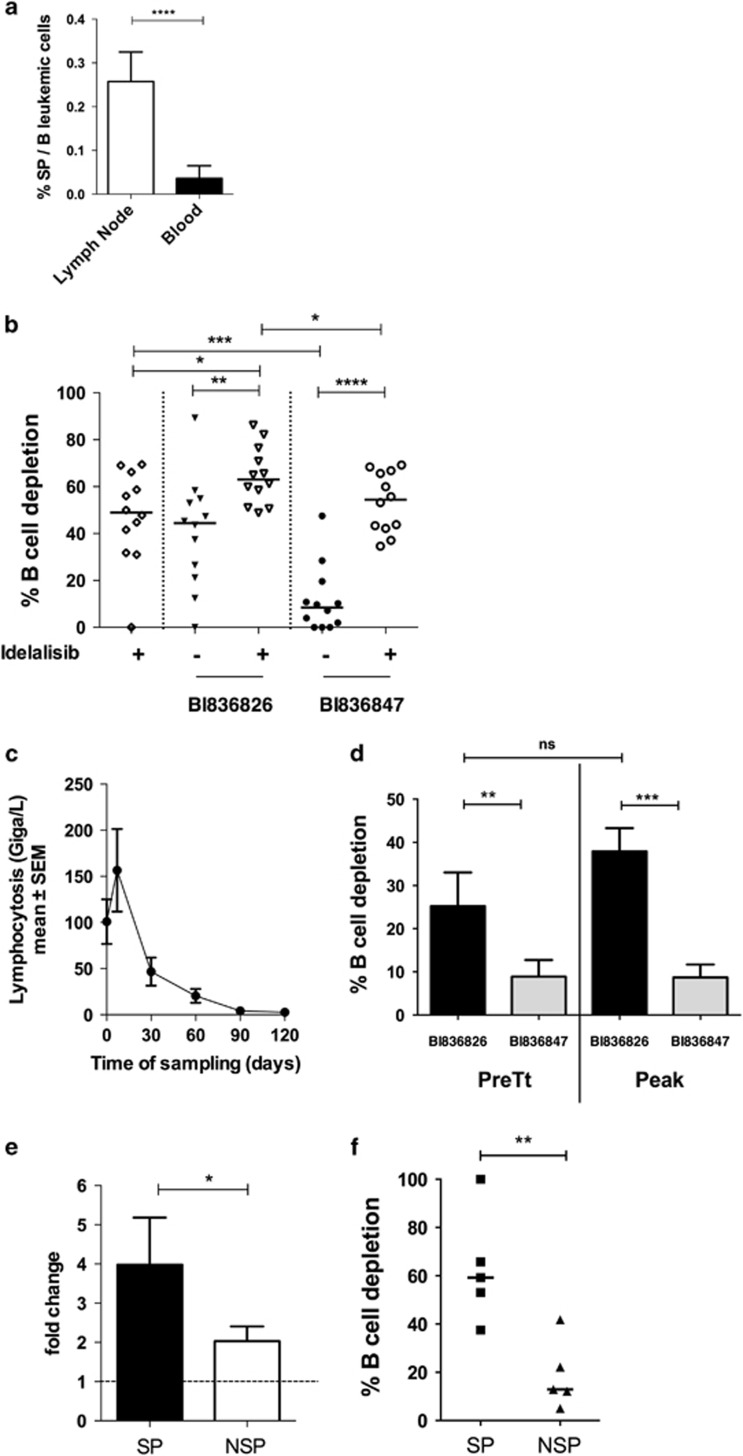
Idelalisib/BI 836826 efficacy in SP and non-SP B leukemic cells from relapsed patients. (**a**) SP cells were analysed as described from frozen lymph node and blood PBMC samples^2^ (*n=*5). (**b**) *In vitro* antibody-induced B-cell depletion: fresh PBMC were incubated with or without Idelalisib (0.5 μM) in the presence BI 836826 or BI 836847 (isotypic control) (10 μg/ml) for 7 days (*n=*12). (**c**) Idelalisib-induced lymphocytosis: blood lymphocytosis was quantified in Idelalisib-treated patients at different time of treatment (*n=*12). (**d**) *In vitro* antibody-induced B-cell depletion at pretreatment and at peak of lymphocytosis (*n=*10). (**e**) Fold change of SP and non-SP in blood samples of Idelalisib treated patients (*n=*5). Fold change= ratio of SP or non-SP absolute number at peak of lymphocytosis *vs* pretreatment. (**f**) *In vitro* antibody-induced B-cell depletion in SP and non-SP populations at peak of lymphocytosis (*n=*5). Experiments were done as described in Figure 1. Flow cytometric assays were done on a BDLSR2 or FORTESSA X20 (Becton Dickinson) and analysed by DIVA software. Statistical analysis: paired Student *t*-test. *P<0.05; **P< 0.001; ***P<0.005; ****P<0.0001.
