# Supplementary material for: Idelalisib improves CD37 antibody BI 836826 cytotoxicity against chemo-resistant /relapse-initiating CLL cells: a rationale for combination treatment
Source: Blood Cancer J. 2016 Nov 11;6(11):e496–. doi: 10.1038/bcj.2016.106 (PMC5148053; doi:10.1038/bcj.2016.106)
Supplement: Supplementary Information [file bcj2016106x1.docx]

**SI 1: Clinical characteristics of relapsed CLL patients**

| *Characteristics* | n |  |
| --- | --- | --- |
|  |  |  |
| Median age (years) | 32 | 69 (49-87) |
| Gender (M/F) | 32 | 22/10 |
| Median leukocytosis per µl | 32 | 95000 |
| FR/FCR treated patients | 22 | 68,75% |
| TT > 2 | 32 | 9,3% |
| IGHV (M/UM) | 26 | 6/20 |
|  |  |  |
| *FISH* |  |  |
| Del13q alone/normal | 23 | 34,7 % |
| Del 11q | 23 | 21,8 % |
| Del 17p | 23 | 4,3 % |
| Tri12 | 23 | 17,4 % |

Abbreviations: M, male; F, female. FR: Fludarabine Rituximab, FCR: Fludarabine Cyclophosphamide Rituximab; TT: treatment; IgVH status: M, mutated; UM, unmutated; del11: 11q deletion; tri12: trisomy12; del13: 13q deletion; del17: 17p deletion,

**SI 2: CD37 expression under Idelalisib treatment**

CD37 expression was analysed by flow cytometry.

(a) *in vitro* Idelalisib treatment (0.5µM) during 7 days; (b) *in vivo* Idelalisb treatment.

**SI 3: Supplemental methods**

**SP and non-SP analysis**

On the basis of the protocol designed by Goodell et al.^9^ primary B-CLL cells were stained with 5 mg/ml of Hoechst 33342 dye (Invitrogen) with or without pre-incubation with ABC transporter inhibitors (Verapamil, 40µM) (Sigma) during 90 min at 37 1C under agitation. Subsequently, cells were incubated on ice with anti-CD19-Alexa700 (Biolegend) and anti-CD5-PE/Cy7 (Biolegend) antibodies or isotype control (Biolegend). Before fluorescent-activated cell sorting analysis, cells were stained with 2 mg/ml of 7-amino-actinomycin D (BD Pharmingen). Data acquisitions were performed on a Fortessa X20 cytometer (BD Bioscience). Flow cytometry acquisitions were analyzed using BD FACSDiva (BD Bioscience). Analysis of SP and non-SP cells was done on the same sample recording 1 million CD19/CD5 events.

**B Cell depletion assays**

According to CLL disease criteria (B leukemic cells > 5G/L in blood) and to provide long-term viability, cultures were performed at high cell density. Fresh PBMC from relapsed CLL patients were seeded at 10 x 10^6^ cells/mL in 10% FCS (de-complemented) culture medium and were either left untreated or treated with saturating dose (10 µg/mL) of control IgG, RTX, BI836826 or BI836847 for 7 days. CD19+/CD5+ (B-leukemic cells) levels were determined by flow cytometry on a BD LSR2 cytometer (BD Bioscience). Specific percentage of remaining B cells in antibodies-treated samples = (Absolute number in treated samples/Absolute number in untreated samples) x 100. For each conditions, absolute number of remaining B cells = total viable cell number (trypan blue exclusion determination) x % of viable CD19+/CD5+ lymphocytes (flow cytometry determination). Then specific B-leukemic cell depletion was calculated as follow: 100−% specific remaining B cells.

For SP and non- SP depletion assays, PBMC were cultured as described and subsequently labeled with Hoechst in the presence or not of Verapamil. Analysis was done as described above.
